# Effect of the combination of bumetanide plus chlorthalidone on hypertension and volume overload in patients with chronic kidney disease stage 4–5 KDIGO without renal replacement therapy: a double-blind randomized HEBE-CKD trial

**DOI:** 10.1186/s12882-022-02930-4

**Published:** 2022-09-20

**Authors:** Fabio Solis-Jimenez, Lucia Monserrat Perez-Navarro, Ricardo Cabrera-Barron, Jesus Antonio Chida-Romero, Geovana Martin-Alemañy, Edgar Dehesa-López, Magdalena Madero, Rafael Valdez-Ortiz

**Affiliations:** 1 Master and Doctorate Program in Health Sciences, Universidad Nacional Autónoma de México, Mexico City, Mexico; 2 Cardiology, Instituto Nacional de Cardiología Ignacio Chváez, Mexico City, Mexico; 3 Nephrology, Hospital General de México Dr. Eduardo Liceaga, Mexico City, Mexico; 4 Nephrology, Hospital Civil de Culiacán, Culiacán, Sinaloa México; 5 Nephrology, Instituto Nacional de Cardiología Ignacio Chávez, Mexico City, Mexico

**Keywords:** Chlorthalidone, Bumetanide, CKD stage 4–5 KDIGO

## Abstract

**Background:**

The co-administration of loop diuretics with thiazide diuretics is a therapeutic strategy in patients with hypertension and volume overload. The aim of this study was to assess the efficacy and safety of treatment with bumetanide plus chlorthalidone in patients with chronic kidney disease (CKD) stage 4–5 KDIGO.

**Methods:**

A double-blind randomized study was conducted. Patients were randomized into two groups: bumetanide plus chlorthalidone group (intervention) and the bumetanide plus placebo group (control) to evaluate differences in TBW, ECW and ECW/TBW between baseline and 30 Days of follow-up. Volume overload was defined as ‘bioelectrical impedance analysis as fluid volume above the 90th percentile of a presumed healthy reference population. The study’s registration number was NCT03923933.

**Results:**

Thirty-two patients with a mean age of 57.2 ± 9.34 years and a median estimated glomerular filtration rate (eGFR) of 16.7 ml/min/1.73 m^2^ (2.2–29) were included. There was decreased volume overload in the liters of total body water (TBW) on Day 7 (intervention: -2.5 vs. control: -0.59, *p* = 0.003) and Day 30 (intervention: -5.3 vs. control: -0.07, *p* = 0.016); and in liters of extracellular water (ECW) on Day 7 (intervention: -1.58 vs. control: -0.43, *p* < 0.001) and Day 30 (intervention: -3.05 vs. control: -0.15, *p* < 0.000). There was also a decrease in systolic blood pressure on Day 7 (intervention: -18 vs. control: -7.5, *p* = 0.073) and Day 30 (intervention: -26.1 vs. control: -10, *p* = 0.028) and in diastolic blood pressure on Day 7 (intervention: -8.5 vs. control: -2.25, *p* = 0.059) and Day 30 (intervention: -13.5 vs. control: -3.4, *p* = 0.018).

**Conclusion:**

In CKD stage 4–5 KDIGO without renal replacement therapy, bumetanide in combination with chlorthalidone is more effective in treating volume overload and hypertension than bumetanide with placebo.

## Introduction

Approximately 85% of patients with advanced chronic kidney disease (glomerular filtration rate < 30 ml/min) suffer from hypertension mostly as a result of volume increase [1]. Given that volume overload is the primary contributing factor to the genesis and maintenance of hypertension in chronic kidney disease patients, loop diuretics have been widely used for treating this subgroup of patients [2]. However, chronic loop diuretic use leads to reduced efficacy over time because cells from the distal convoluted tubule undergo hypertrophy in response to the abnormally high sodium concentrations resulting from blockage of the Na–K-Cl cotransporter (NKCC) in Henle’s loop, facilitating the distal reabsorption of sodium and chlorine [3]. This phenomenon is known as resistance to loop diuretics and is manifested by the presence of volume overload and arterial hypertension despite the chronic use of loop diuretics [4]. One approach to overcome loop diuretic resistance is the addition of a thiazide-type diuretic to produce diuretic synergy via "sequential nephron blockade," first described more than 40 years ago in patients with heart failure [5]; however, some studies that show the potential positive effect of adding a thiazide diuretic in patients with chronic kidney disease reinforce the need for a randomized trial to demonstrate the safety and efficacy of thiazides in advanced chronic kidney disease [6, 7]. Thiazide diuretics decrease blood pressure by blocking the thiazide-sensitive Na + -Cl − symporter, preventing reabsorption of sodium (Na +) and chloride (Cl −) ions from the distal convoluted tubule. When this occurs, NaCl and water are released to the lumen, and the amount of urine produced per day increases; however, their use is not recommended in advanced chronic kidney disease due to their suspected lack of natriuretic and antihypertensive effects when the glomerular filtration rate falls below 30 ml/min [7]. This notion has been recently disputed, as the effect of hydrochlorothiazide has been recognized in patients with advanced chronic kidney disease [8]. In the same way, the CLICK study was recently published, which showed the efficacy of chlorthalidone in controlling blood pressure in patients with CKD stage 4 KDIGO [9]. The aim of our study was to evaluate the additional effect of co-administration of bumetanide with chlorthalidone on blood pressure and volume overload in patients with advanced chronic kidney disease and chronic loop diuretic treatment and without renal replacement therapy.

## Material and methods

### Type of study

A single-center, randomized, double-blind (nephrologist and patient), parallel, 1:1 allocation ratio, bumetanide plus placebo (control group) trial was conducted to compare bumetanide plus chlorthalidone (intervention group) in patients with advanced chronic kidney disease (KDIGO stages 4 and 5) with hypertension, volume overload and chronic loop diuretic use. We defined chronic loop diuretic use as patients with at least three consecutive months of furosemide (at least 40 mg daily) or bumetanide (at least 1 mg daily) use. Bumetanide [10, 11] and chlorthalidone [12, 13] doses were based on pharmacokinetic studies of diuretics in patients with chronic kidney disease. Likewise, the selection of bumetanide (as a loop diuretic) and chlorthalidone (as a thiazide diuretic) was made based on the availability of both drugs in our institution. The study consisted of an initial screening evaluation, followed by a follow-up evaluation on Day 30. The screening and enroll period was from June 18, 2019 to October 28, 2019. During the initial screening, the medical history was recorded and a physical examination was performed, including anthropometric measurements, bioelectrical impedance analysis, and blood pressure, to evaluate subject eligibility.

### Study design

Randomized groups were assigned to receive initial treatment with either 3 mg bumetanide + 50 mg chlorthalidone or 3 mg bumetanide + placebo. Bumetanide was administered on the following schedule: 2 mg at 10 am and 1 mg at 4 pm. This bumetanide dose schedule was defined according to the experience in the use of bumetanide from our department and based on pharmacokinetic studies [10–13]. Chlorthalidone was administered on the following schedule: 50 mg at 12 pm. The second assessment was performed 7 days after the start of the clinical trial and included new blood tests, blood pressure measurements, bioelectrical impedance analysis, and questionnaires for possible adverse effects. If no contraindication was noted [symptomatic hypotension secondary to clinical evidence of dehydration; severe hyponatremia (sodium < 128 mEq/L); or severe hypokalemia (potassium < 3.5 mEq/L)], the treatment dosage was escalated to 4 mg bumetanide + 100 mg chlorthalidone (intervention group) or bumetanide 4 mg + placebo (control group). The placebo consisted of starch capsules, which had the same appearance and weight as the chlorthalidone capsules. Twenty-eight days after the start of the initial evaluation, patients were subjected to the final assessment, which consisted of blood testing, bioelectrical impedance analysis, blood pressure measurements, and a questionnaire.

### Selection criteria

The inclusion criteria were uncontrolled hypertension (systolic blood pressure (SBP) > 140 mmHg and/or diastolic blood pressure (DBP) > 90 mmHg) despite antihypertensive treatment, volume overload (defined by bioelectrical impedance analysis as fluid volume above the 90th percentile of a presumed healthy reference population), chronic loop diuretic use (at least three months), chronic kidney disease stages 4 or 5 diagnosed at least three months previously, age between 18 and 75 years, and a signed informed consent form. The exclusion criteria were contraindications for chlorthalidone use (known allergy to thiazide diuretics), pregnancy, breastfeeding, cognitive deterioration, non-steroidal anti-inflammatory drug (NSAID) use, acute heart failure, chronic liver failure, respiratory insufficiency, and any cancer. Eligible patients were recruited from the nephrology clinic of our hospital and scheduled for the initial consultation of the treatment period, which consisted of basal blood testing to screen for treatment contraindications, as well as the estimated initial glomerular filtration rate by CKD-EPI; the etiology of chronic kidney disease was attributed to hypertension or diabetes in the case of having these long-standing diseases, without clinical evidence of another contributing factor. One patient with a biopsy showing lupus nephropathy participated in the clinical trial, and those patients without comorbidities and without a biopsy showing the etiology of kidney disease were classified as of unknown cause. Bioelectrical impedance analysis was also performed at this time using a seca mBCA 514 medical Body Composition Analyzer (Hamburg, Germany). Blood pressure measurements were performed by trained physicians blinded to the treatment group under the 2018 ESC/ESH guidelines for the management of arterial hypertension using a mercury sphygmomanometer of adequate size (12 × 22 cm^2^, 16 × 30 cm^2^, or 16 × 36 cm^2^).

### Primary and secondary outcomes

The primary outcome was to evaluate differences in total body water (TBW), extracellular water (ECW) and ECW/TBW between baseline and Days 7 and 30 of follow-up. Electrical bioimpedance vector analyses were performed to evaluate these primary outcomes. Secondary outcomes included differences in baseline SBP, DBP, mean arterial pressure (MAP), fraction of sodium excreted (FENA), and brain natriuretic peptide (BNP) measurements compared with Days 7 and 30 of follow-up.

### Safety outcomes

The following effect outcomes were recorded: impaired kidney function (defined as an increase in creatinine serum ≥ 0.3 mg/dL from baseline); creatinine doubling (compared with baseline creatinine); hyponatremia (serum sodium values ≤ 128 mEq/L); hypokalemia (serum potassium ≤ 3.5 mEq/L); hyperuricemia (serum uric acid ≥ 8 mEq/L); cardiovascular events (acute myocardial infarction, hospitalization for heart failure, or stroke); and mortality for all causes or cardiovascular causes.

### Statistical analysis

A double-blind (nephrologist and patient) method was utilized, whereby the patient and researcher were unaware of allocations to the bumetanide plus placebo and treatment groups. As part of the statistical plan, an Excel database was created in which the content of the data collection sheets was captured. The information was later exported to the STATA 14 statistical program (Computing Resource Center in California). A statistical power analysis was performed to estimate sample size based on data from the study of chlorthalidone effects on blood pressure, pulse and weight in patients with low kidney function [13]. With an alpha = 0.05 and power = 0.80, the projected sample size needed with this effect size was calculated in GPower 3.1.9.2, resulting in *n* = 32 for this simple comparison between groups. Measurements were compared at the various time points. Randomization was performed by the nephrology research director using a simple 1:1 model and online software (https://www.graphpad.com/quickcalcs/randomize1.cfm). Parametric and nonparametric statistical tests were performed depending on the distribution of variables for the evaluation of each of the primary and secondary outcomes. The mean and standard deviation are shown for variables with a normal distribution, and the median and range are shown for variables without a normal distribution. Effect size was compared with Cohen's D test between groups for body water parameters (TBW, ECW and ECW/TBW); blood pressure (SBP, DBP and MAP); and of the changes of FENA and BNP. A *p* value < 0.05 was considered statistically significant.

## Results

**Fig. 1:**
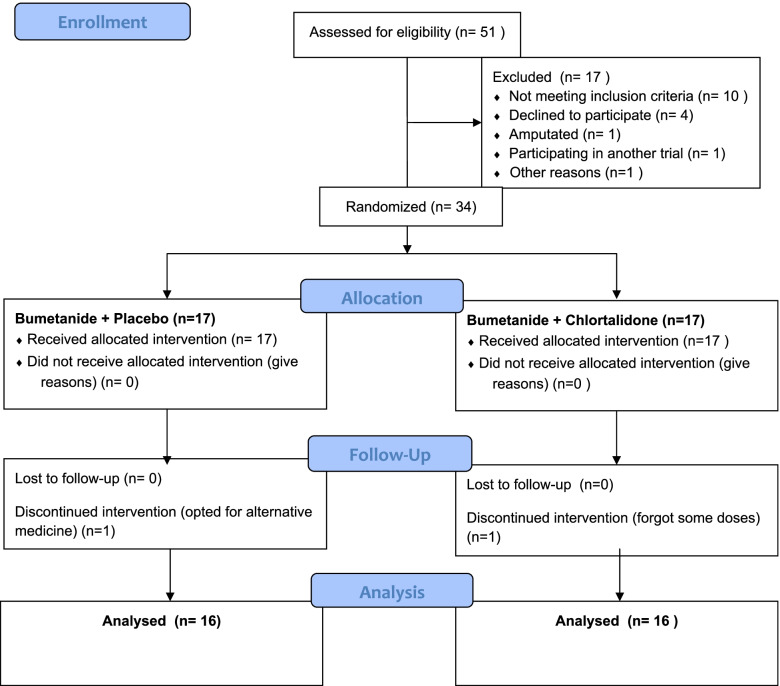
CONSORT 2010 flow diagram

Fifty-one patients were assessed for eligibility, and 34 were randomized (17 in each group). Finally, sixteen participants from each group were included in the analysis. Two participants (one from each group) were excluded from the analysis because they did not complete the follow-up. The recruitment period lasted 3 months (Fig. 1).

### Descriptive statistics

The mean subject age was 57.2 ± 9.34 years, 22 subjects were female (68.8%), the mean time from CKD diagnosis was 24 months (3–120), 68.8% were diabetic, the median diuretic use was 12 months (3–60), and furosemide was the most common loop diuretic (93.8%). The median glomerular filtration rate was 16.7 ml/min/1.73 m^2^ (2.2–29), and 17 patients (53.1%) were in KDIGO stage 4. No significant differences in baseline characteristics were observed between groups (Table 1).

**Table 1:** Baseline characteristics of the study participants

Variables	Total *n* = 32	Bumetanide plus chlorthalidone *n* = 16	Bumetanide plus placebo *n* = 16	*p*
Age *X* ± *SD* (*years*)	57.2 ± 9.34	54.8 ± 10	59.6 ± 8.1	0.148
Gender *Women* (%)	22 (68.8)	10 (62.5)	12 (75)	0.462
Weight *X* ± *SD (kg)*	71.3 ± 12.7	74.6 ± 14.4	67.9 ± 10.1	0.139
BMI *X* ± *SD (kg/m*^*2*^*)*	29.1 ± 4.2	30.1 ± 4.9	28.1 ± 3.1	0.181
**CKD etiology (%)**				0.919
Diabetes	22 (68.7)	11 (68.7)	11 (68.7)	
Unknown	7 (21.8)	4 (25)	3 (18.7)	
Hypertension	2 (6.25)	1 (6.3)	1 (6.3)	
Others	1 (3.25)	0 (0)	1 (6.3)	
**Comorbidities (%)**				0.753
Hypertension	32 (100)	16 (100)	16 (100)	
Smoking	15 (46.9)	7 (43.8)	8 (50)	
Diabetes	22 (68.8)	11 (68.8)	11 (68.8)	
Lupus	1 (3.1)	0 (0)	1 (6.3)	
**Body Water by Bioimpedance**				
TBW *median* ± *range (liters)*	33.1 (20–70.6)	32.7 (23.4–70.6)	33.1 (20–53.7)	0.205
ECW *median* ± *range (liters)*	17.7 (10.7–35.7)	16.4 (12.8–35.7)	16.2 (10.7–25.8)	0.233
ECW/TBW *median* ± *range* (*%*)	50.4 ± 3.5	50 ± 3.6	50.9 ± 3.5	0.509
**Systemic Blood Pressure** (*mmHg*)				
SBP *X* ± *DE*	144.6 ± 20.3	142 ± 22.6	146.8 ± 18.2	0.546
DBP *X* ± *DE*	79.7 ± 11.1	81 ± 10.9	77.8 ± 11.3	0.348
MAP X ± *DE*	101 ± 12.7	102.1 ± 10.9	100.6 ± 12.8	0.756
**Laboratory tests**				
Creatinine *median, CI95% (mg/dL)*	3.6 (1.83–16.3)	3.6 (1.8–16.6)	3.5 (1.9–15)	0.759
Urea *median, CI95% (mg/dL)*	125.4 (57.9–269.8)	125 (57.9–244.9)	124(85.4–269)	0.916
GFR median, CI95% *(ml/min/1.73 m*^*2*^*)*	16.1 ± 8.14	16.52 ± 8.76	15.69 ± 7.64	0.778
Serum sodium *X* ± *DE (mEq/l)*	137.9 ± 4.6	137.4 ± 4.9	138.3 ± 4.3	0.616
Serum potassium *X* ± *DE (mEq/l)*	5.2 ± 0.68	5.3 ± 0.64	5.1 ± 0.74	0.614
Serum bicarbonate *X* ± *DE (mEq/l)*	18.5 (14.1–24.6)	18.4 (14.2–23.7)	18.5 (14.1–24.6)	0.947
Serum BNP *median* ± *range (pg/mL)*	78.5 (10–960)	67.1 (10–960)	112(24.7–424-6)	0.694
Serum uric acid *median* ± *range (mg/dL)*	7 (3.4–14.1)	6.6 (3.1–14.1)	7.3 (3.6–11.6)	0.772
Serum albumin *median* ± *range (g/L)*	3.8 (1.8–4.75)	3.8 (1.78–4.4)	3.8 (2.9–5)	0.265
Urinary Sodium *X* ± *SD (mEq/l)*	63.7 ± 21.6	62.7 ± 20.6	64.7 ± 23.4	0.800
Urinary Chlorine *X* ± *SD (mEq/l)*	61.2 ± 23.1	62.3 ± 18.6	60.2 ± 27.4	0.798
* Fraction of sodium excreted, median* ± *range (%)*	4.2 (0.9–17.9)	3.6 (1.4–8.42)	4.8 (0.9–17.9)	0.332
**24-h Urine Volume** *X* ± *SD (ml)*	1739 ± 696	1832 ± 729	1646 ± 673	0.458
**Antihypertensive Drugs (%)**				
Angiotensin Converting Enzyme-Inhibitor	6 (18.75)	2 (12.5)	4 (25)	0.381
Angiotensin Receptor Blocker	23 (71.87)	11 (68.75)	12 (75)	0.705
Alfa Blocker	9 (28.12)	5 (31.25)	4 (25)	0.705
Beta Blocker	11 (34.37)	6 (37.5)	5 (31.25)	0.721
Calcium Channel Blocker	26 (81.25)	14 (87.5)	12 (75)	0.381
**Number antihypertensive Drugs (%)**				
1	1 (3.12)	0 (0)	1 (6.3)	0.325
2	10 (31.2)	6 (37.5)	4 (25)	0.462
≥ 3	21 (65.6)	10 (62.5)	11 (68.7)	0.462
**Type of loop diuretic (%)**				0.154
Furosemide	30 (93.8)	14 (87.5)	16 (100)	
Bumetanide	2 (6.3)	2 (12.5)	0 (0)	
**Time on loop diuretic** (median ± range) (months)	12 (2–60)	9 (2–60)	15 (2–36)	0.939

*Abbreviations*: *BMI* Body mass index, *CKD* Chronic Kidney Disease, *TBW* Total Body Water, *ECW* Extracellular Water, *SBP* Systolic blood pressure, *PAD* Diastolic blood pressure, *MAP* Mean Arterial Pressure, *BNP* Brain natriuretic peptide

### Primary outcome

**Fig. 2:**
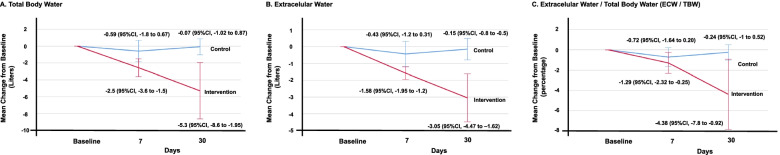
Total body water analysis by bioimpedance vectors. The bumetanide plus placebo group is shown in blue (control group), and the bumetanide plus chlorthalidone group is shown in red (intervention group). **A** There was decreased volume overload in liters of TBW on Day 7 [intervention: -2.5 (95% CI, -3.6 to -1.5) vs. control: -0.59 (95% CI, -1.8 to 0.67), *p* = 0.003] and Day 30 [intervention: -5.3 (95% CI, -8.6 to -1.95) vs. control: -0.07 (95% CI, -1.02 to 0.87) *p* = 0.016]. **B** There was a decrease in ECW on Day 7 [intervention: -1.58 (95% CI, -1.95 to -1.2) vs. control: -0.43 (95% CI, -1.2 to 0.31) *p* < 0.001] and Day 30 [intervention: -3.05 (95% CI, -4.47 to –1.62) vs. control: -0.15 (95% CI, -0.8 to -0.5), *p* < 0.000]. **C** There was a reduction in the ECW/TBW ratio on Day 7 [intervention: -1.29 (95% CI, -2.32 to -0.25) vs. control: -0.72 (95% CI, -1.64 to 0.20), *p* = 0.384] and Day 30 [intervention: -4.38 (95% CI, -7.8 to -0.92) vs. control: -0.24 (95% CI, -1 to 0.52), *p* = 0.018]

There was a decrease in volume overload in the liters of TBW on Day 7 [intervention: -2.5 (95% CI, -3.6 to -1.5) vs. control: -0.59 (95% CI, -1.8 to 0.67), *p* = 0.003] and Day 30 [intervention: -5.3 (95% CI, -8.6 to -1.95) vs. control: -0.07 (95% CI, -1.02 to 0.87) *p* = 0.016]; in liters of ECW on Day 7 [intervention: -1.58 (95% CI, -1.95 to -1.2) vs. control: -0.43 (95%CI, -1.2 to 0.31), *p* < 0.001], and Day 30 [intervention: -3.05 (95% CI, -4.47 to –1.62) vs. control: -0.15 (95%CI, -0.8 to -0.5), *p* < 0.000]; and in the ECW/TBW ratio on Day 7 [intervention: -1.29 (95% CI, -2.32 to -0.25) vs. control: -0.72 (95% CI, -1.64 to 0.20), *p* = 0.384] and Day 30 [intervention: -4.38 (95% CI, -7.8 to -0.92) vs. control: -0.24 (95% CI, -1 to 0.52), *p* = 0.018] (Fig. 2A-C).

### Secondary outcomes

**Fig. 3:**
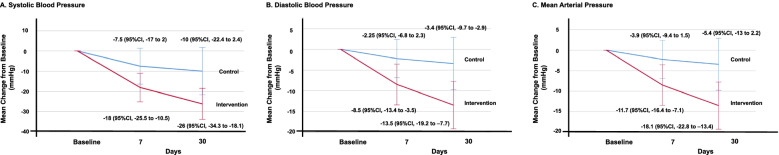
Effects on blood pressure. The bumetanide plus placebo group is shown in blue (control group), and the bumetanide plus chlorthalidone group is shown in red (intervention group). **A** There was a decrease in SBP on Day 7 [intervention: -18 (95% CI, -25.2 to -10.5) vs. control: -7.5 (95% CI, -17 to 2), *p* = 0.073] and Day 30 [intervention: -26.1, 95% CI, -34.3 to -18.1, vs. control: -10 95% CI, -34.3 to -18.1; *p* = 0.028]. **B** There was a reduction in DBP on Day 7 (intervention: -8.5, 95% CI, -13.4 to -3.5, vs. control: -2.25, 95% CI, -6.8 to 2.3, *p* = 0.059) and Day 30 [intervention: -13.5 (95% CI, -19.2 to -7.7) vs. control: -3.4 (95% CI, -9.7 to -2.9), *p* = 0.018]. **C** There was a decrease in MAP on Day 7 (intervention: -11.7, 95% CI, -16.4 to -7.1, vs. control: -3.9, 95% CI, -9.4 to 1.5, *p* = 0.029) and Day 30 (intervention: -18.1, 95% CI, -22.8 to -13.4, vs. control: -5.4 95% CI, -13 to 2.2, *p* = 0.005)

There was a decrease in SBP on Day 7 [intervention: -18 (95% CI, -25.2 to -10.5) vs. control: -7.5 (95% CI, -17 to 2), *p* = 0.073] and Day 30 [intervention: -26.1, (95% CI, -34.3 to -18.1) vs. control: -10 (95% CI, -34.3 to -18.1), *p* = 0.028]; in the DBP on Day 7 [intervention: -8.5, (95% CI, -13.4 to -3.5) vs. control: -2.25, (95% CI, -6.8 to 2.3), *p* = 0.059) and Day 30 [intervention: -13.5 (95% CI, -19.2 to -7.7) vs. control: -3.4 (95% CI, -9.7 to -2.9) *p* = 0.018]; and in the MAP on Day 7 [intervention: -11.7, 95% CI, -16.4 to -7.1, vs. control: -3.9 95% CI, -13 to 2.2, *p* = 0.029] and on Day 30 [intervention: -18.1 (95% CI, -22.8 to -13.4) vs. control: -5.4 (95% CI, -13 to 2.2), *p* = 0.005], see Fig. 3 A-C. Along this same line, a significantly lower requirement for use of equal or more than three antihypertensives (other than chlorthalidone) was observed in the group with bumetanide and chlorthalidone (baseline: 62.5% vs. final: 25%, *p* = 0.009); but not in patients with bumetanide and placebo (baseline: 68.8% vs. final: 87.5%, *p* = 0.083).

**Fig. 4:**
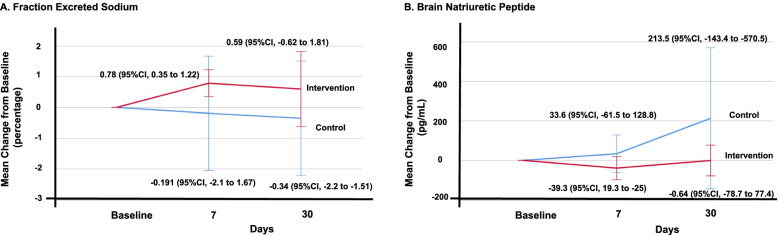
Effect on fractional sodium excretion (FENa) and brain natriuretic peptide (BNP). The bumetanide plus placebo group is shown in blue (control group), and the bumetanide plus chlorthalidone group is shown in red (intervention group). **A** Analysis of FENA revealed that during the first week after the intervention, the bumetanide plus chlorthalidone group showed a decrease in FENA from 0.78 (95% CI, 0.35 to 1.22) to 0.59 (95% CI, -0.62 to 1.81) without intragroup statistical significance (*p* = 0.104), while the bumetanide plus placebo group had a change from baseline to day 30 of -0.34 (95% CI, -2.2 to -1.51; *p* = 0.605). **B** At the start of follow-up, the bumetanide plus placebo group showed an increase in BNP on Day 7 of 33.6 (95% CI, -61.5 to 128.8 pg/dL) and on Day 30 of 213.5 (95% CI, -143.4 to -570.5 pg/dL) without statistical significance (*p* = 0.175). The intervention group showed a decrease on Day 7 of -39.3 (95% CI, 19.3 to -25 pg/dL) and on Day 30 of -0.64 (95% CI, -78.7 to 77.4 pg/dL) without a significant difference (*p* = 0.221). Similarly, intergroup analysis did not reveal any significant differences

Analysis of FENA revealed that during the first week after the intervention, the bumetanide plus chlorthalidone group showed a decrease in FENA from 0.78 (95% CI, 0.35 to 1.22%) to 0.59 (95% CI, -0.62 to 1.81%), while the bumetanide plus placebo group showed a change from baseline to day 30 of -0.34 (95% CI, -2.2 to -1.51%), both groups without intragroup statistical significance (*p* = 0.104 and *p* = 0.605, respectively) (Fig. 4A). Likewise, the comparison between the control and intervention groups did not show a significant difference at either 7 days (*p* = 0.284) or at 30 days (*p* = 0.371). Brain natriuretic peptide levels were also evaluated. At the start of follow-up, the bumetanide plus placebo group showed an increase in BNP on Day 7 to 33.6 (95% CI, -61.5 to 128.8 pg/dL) and on Day 30 to 213.5 (95% CI, -143.4 to -570.5 pg/dL) without statistical significance (*p* = 0.175). The intervention group showed a decrease of -39.3 on Day 7 (95% CI, 19.3 to -25 pg/dL) and -0.64 on Day 30 (95% CI, -78.7 to 77.4 pg/dL) without a significant difference (*p* = 0.221). Similarly, intergroup analysis did not reveal any significant differences (Fig. 4B).

Effect size analysis is shown in Table 2. Large effect sizes (*d* greater than 0.5) were observed for changes in blood pressure (SBP, DBP, and MAP) and changes in body water (TBW, ECW and ECW/TBW).

**Table 2:** Effect size (Cohen’s D) between groups

Variables	**Bumetanide plus placebo** Mean ± SD	**Bumetanide plus chlorthalidone** Mean ± SD	***p*-value**	**Cohen's D**
**Changes of SBP**				
7 day	-7.5 ± 17.8	-18.1 ± 14.1	0.073	0.66
30 day	-10 ± 23.3	-26.2 ± 15.3	0.028	0.84
**Changes of DBP**				
7 day	-2.25 ± 8.6	-8.5 ± 9.3	0.059	0.69
30 day	-3.40 ± 11.9	-13.5 ± 10.7	0.018	0.89
**Changes of MAP**				
7 day	-3.97 ± 10.2	-11.7 ± 8.7	0.029	0.82
30 day	-5.41 ± 14.3	-18.1 ± 8.7	0.005	1.10
**Changes of TBW**				
7 day	-0.59 ± 2.38	-2.5 ± 1.9	0.016	0.89
30 day	-0.07 ± 1.78	-5.3 ± 6.2	0.003	1.31
**Changes of ECW**				
7 day	-0.43 ± 1.4	-1.58 ± 0.69	0.007	1.10
30 day	-0.15 ± 1.2	-3.11 ± 2.68	< 0.000	1.52
**Changes of ECW/TBW**				
7 day	-0.72 ± 1.7	-1.24 ± 1.94	0.077	1.21
30 day	-0.24 ± 1.4	-4.38 ± 6.49	0.018	1.05
**Changes of FENA**				
7 day	-0.19 ± 3.4	0.78 ± 0.82	0.284	0.28
30 day	-0.35 ± 3.4	0.59 ± 2.29	0.371	0.08
**Changes of BNP**				
7 day	33.6 ± 178	-39.3 ± 110	0.174	0.04
30 day	213.5 ± 669	-0.64 ± 146	0.221	0.52

*Abbreviations*: *SBP* Systolic Blood Pressure, *PAD* Diastolic Blood Pressure, *MAP* Mean Arterial Pressure, *TBW* Total Body Water, *ECW* Extracellular Water, *ECW/BTW* Total Body Water/Extracellular Water Ratio, *FENA* Fraction Excretion Sodium, *BNP* Brain Natriuretic Peptide

### Safety analysis

**Fig. 5:**
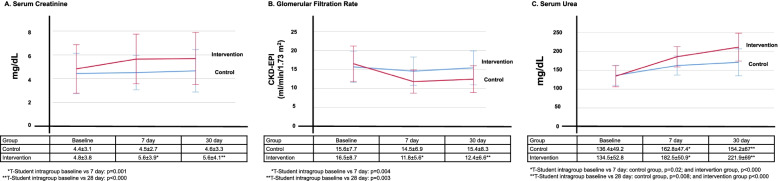
Effect of treatment on renal function. The bumetanide plus placebo group is shown in blue (control group), and the bumetanide plus chlorthalidone group is shown in red (intervention group). **A** Regarding serum creatinine, the intervention group showed a significant increase at Day 7 (*p* = 0.001) and Day 30 (*p* < 0.000). **B** Regarding GFR, a significant decrease in the intervention group was observed at Day 7 (*p* = 0.004) and Day 30 (*p* = 0.003). **C** Serum urea levels in both groups showed significant increases when comparing baseline versus Days 7 and 30 (control group, *p* = 0.02 and *p* < 0.000, respectively, and intervention group, *p* = 0.008 and *p* < 0.000, respectively)

The most common adverse event observed in this study was increased creatinine levels (Cr > 0.3 mg/dL), which were present in eleven cases with a range of 2.63 mg/dL (68.75%), compared with four cases in the bumetanide plus placebo group with a range of 1.86 mg/dL (25%) (*p* = 0.013). Nevertheless, none of the patients exhibited a two-fold increase in their serum creatinine levels at the end of follow-up, nor did they require renal replacement therapy. Other adverse effects are listed in Table 3. Figure 5 shows a comparison of changes in serum creatinine (Fig. 5A), glomerular filtration rates (Fig. 5B), and serum urea levels (Fig. 5C) during the study. Regarding serum creatinine levels, the intervention group showed a significant increase at seven (*p* = 0.001) and twenty-eight days (*p* < 0.000). In this same group, a significant decrease in the glomerular filtration rate was observed at seven (*p* = 0.004) and twenty-eight days (*p* = 0.003). Regarding urea levels, both groups showed significant increases when comparing baseline with Days 7 and 30. The comparison of serum levels of creatinine and urea and the glomerular filtration rate estimated by CKD-EPI between the control and intervention groups did not show significant differences. During the follow-up time of the clinical trial patient, none started renal replacement therapy and none presented clinical data of volume depletion.

**Table 3:** Proportions of patients with adverse events during the study period

Adverse events (%)	Bumetanide plus placebo *n* = 16	Bumetanide plus chlorthalidone *n* = 16	*p*
Impaired kidney function	4 (25)	11 (68.75)	0.013
Creatinine doubling	0 (0)	0 (0)	-
Hyponatremia	1 (6)	2 (12)	1.000
Hypokalemia	0 (0)	2 (12)	0.484
Hyperuricemia	4 (25)	4 (25)	1.000
Cardiovascular events	0 (0)	1 (6)	1.000
Mortality	0 (0)	0 (0)	-

## Discussion

Our results demonstrated the effect of combined bumetanide and chlorthalidone therapy in patients with advanced chronic kidney disease. Among the significant findings, a direct effect of combined therapy with respect to total body water levels and blood pressure was found throughout the study. Trials up to the time of this study evaluating the efficacy and safety of bumetanide plus chlorthalidone treatment with diuretics in patients with chronic kidney disease are limited [6]. Current KDIGO guidelines do not recommend thiazide diuretic use in advanced chronic kidney disease, arguing that the efficacy of this type of diuretic diminishes proportionally with the glomerular filtration rate, suggesting the substitution of thiazide diuretics with a loop diuretic when the glomerular filtration rate dips below 30 ml/min [7]. This recommendation originated from a study conducted in 1961, in which a poor natriuretic effect of thiazides was observed in two patients with glomerular filtration rates below 15 ml/min [14]. Hoshino et al. found that the combination of hydrochlorothiazide and loop diuretics improved blood pressure levels and decreased proteinuria in eleven advanced stage type 2 DKD patients with severe edema [15]. Previously, in the nineties, Fliser et al. showed that the combination of torsemide with a thiazide diuretic significantly increased urinary sodium and chloride elevation in ten patients [16]. Recently, Agarwal et al. showed in an excellent clinical trial the positive impact on blood pressure of adding chlorthalidone in patients with stage 4 CKD. This trial compared the use of chlorthalidone vs placebo in patients with stage 4 chronic kidney disease and poorly controlled hypertension. A reduction of 10 mmHg in 24-h systolic blood pressure in patients who were treated with chlorthalidone was found, even if they were previously treated with loop diuretics, although these patients had a higher risk of significantly increasing serum creatinine levels [9]. In our study, the mean blood pressure reduction was found to be higher than the results observed in other trials. This may be due to the important role of volume overload on hypertension in patients with chronic kidney disease. There is evidence in patients undergoing hemodialysis that less than 5% of patients who are in dry weight, maintain a systolic pressure above 140 mmHg [17]. Agarwal et al. also demonstrated a significant decrease in systolic and diastolic pressure in patients who achieved dry weight [18]. All the patients included in this study suffered from significant volume overload before being randomized to the study, so it is likely that phenomenon that probably influenced our outcomes. The results obtained in our study not only demonstrate the antihypertensive effect of thiazide diuretics in advanced chronic kidney disease but also demonstrate the benefit of combined therapy in treating volume overload.

Our findings could have a positive clinical impact in management of volume overload in the chronic kidney disease population, whose prognosis has already been proven to improve with a reduction in volume overload. *Szu-Chun Hung* et al. employed a model to predict mortality based on patients’ extracellular water to total body water ratio. This ratio expresses the amount of extracellular water that exists compared to total body water. In healthy people, the index is between 0.36 and 0.39, however, it has been observed that it is increased in patients with advanced chronic kidney disease and has been associated with multiple adverse outcomes. Patients were classified into two groups based on the extracellular water to total body water ratio with a cutoff of ± 48%, and after four years of follow-up, they observed twice the mortality rate in those with levels ≥ 48% [19]. These results could be of particular interest when applied to this trial since, before the intervention, both groups had an ECW/TBW ratio > 50%; however, at the end of follow-up, only the combined treatment group exhibited a reduction to 46%, which could be impactful as a possible benefit in terms of mortality in this population.

Another finding was that treatment with loop diuretics alone was insufficient for blood pressure and volume overload control compared with bumetanide plus chlorthalidone. This was evidenced by the fact that in our trial, all patients had a history of chronic loop diuretic use (most were on furosemide); however, when they were randomized, furosemide was changed to bumetanide, a more potent loop diuretic with a dose equivalent to 160 mg furosemide QD [20]. This protocol, along with our findings, allowed us to demonstrate that high doses of loop diuretics are not sufficient by themselves to achieve adequate blood pressure and volume overload in patients with advanced chronic kidney disease without renal replacement therapy. When employing high doses of bumetanide, the possibilities of poor efficacy of the loop diuretic secondary to pharmacokinetic processes associated with diuretic resistance, such as insufficient dosage, reduced absorption, low bioavailability, and interaction with uremic toxins, are diminished [21]. We consider that this difference could be explained by chronic loop diuretic use and previously described adaptive changes at the distal convoluted tubule, in which patients exhibit higher sodium reabsorption, lower natriuresis, and, as a consequence, loss of loop diuretic efficacy [22, 23].

Thiazide diuretic use, particularly chlorthalidone, seems to provide additional benefits; chlorthalidone has a potency at least threefold that of hydrochlorothiazide, as well as a longer half-life [24]. A recent meta-analysis demonstrated that chlorthalidone has a larger antihypertensive effect than hydrochlorothiazide with a comparable safety profile [25]. Other studies have demonstrated that chlorthalidone may be superior to hydrochlorothiazide with respect to inducing regression of left ventricular hypertrophy and preventing cardiovascular events [26, 27]. As described in other studies, our results suggest that the effect of bumetanide plus chlorthalidone therapy on blood pressure levels could be related to the natriuretic effect with subsequent volume depletion produced by thiazide diuretics [28]. Even studies conducted in anuric patients failed to demonstrate an antihypertensive effect of thiazides independent of natriuresis and volume depletion [29]. Regarding the dose of chlorthalidone used in our study, we recognize that these doses are higher than the 12.5 to 25 mg/day dose that is usually recommended in patients with arterial hypertension [30]; however, doses > 50 mg (optimal doses) have been evaluated and defined in the context of high-dose diuretic therapy, as derived from studies that evaluated the dose–response curve of thiazide diuretics [31]. The pharmacokinetics of chlorthalidone doses between 50 and 200 mg reveal optimal erythrocyte half-life values and urinary excretion in patients with CKD [12]. In addition, we considered pharmacokinetic adjustment according to the deterioration in the glomerular filtration rate present in our patients to achieve the expected natriuretic effect [13]. We must recognize that there are no update pharmacokinetic studies of chlorthalidone in patients with advanced chronic kidney disease, which represents a challenge and a weakness of our study. Based on the above and based on the results of our work, it would be important to design pharmacokinetic studies of chlorthalidone with or without loop diuretics in patients with advanced chronic kidney disease as a future area of research.

Actual estimates indicate that nearly 40% of patients diagnosed with advanced chronic kidney disease decide not to start renal replacement therapy, choosing conservative treatment instead [32]. Our results support the notion that bumetanide plus chlorthalidone therapy could be an alternative in patients with advanced chronic kidney disease who have not yet accepted the initiation of renal replacement therapy to reduce volume overload and optimize blood pressure control, with the aim of achieving a better quality of life. However, this pharmacological therapy with diuretics will always be limited by the prerequisite of residual uresis in patients subject to this treatment modality.

On the other hand, BNP has been correlated with volume overload in patients with chronic kidney disease measured by bioimpedance [33, 34]; however, in our trial, BNP levels did not behave in a manner that reflected a reduction in volume overload, as evidenced by bioimpedance vector analysis. This result can be explained by results obtained in other studies in patients subject to renal replacement therapy in which it was demonstrated that a significant reduction in BNP levels (< 100 pg/dL, only attainable after multiple hemodialysis sessions) is associated with ECW/TBW ratios < 40% [35, 36]. For this reason, we consider that even though our patients exhibited decreased ECW/TBW ratio, at the end of follow-up, none achieved the ideal dry weight. Therefore, the reduced volume overload observed in the bumetanide plus chlorthalidone group was not sufficient to diminish cardiac muscle stress or achieve a significant reduction in BNP levels.

We observed the same phenomenon in plasma urea levels and glomerular filtration rate (GFR), both associated with a nonsignificant deterioration in patients on bumetanide plus chlorthalidone treatment compared with bumetanide plus placebo. The proportion of patients in the bumetanide plus chlorthalidone group who experienced an increase in plasma creatinine levels could be conceptually defined as having acute kidney injury [37]. Nevertheless, grading by AKIN or RIFLE classifications (utilized for acute kidney failure diagnosis) has not been applied in advanced chronic kidney disease populations [38]. Among the reasons for not applying these criteria to diagnose acute kidney failure in patients with advanced chronic kidney disease and chronic diuretic use is the fact that urinary volume measurements, considered part of the diagnostic criteria for acute kidney failure, cannot be applied because urinary volume in this population is variable and dependent on residual renal tubular function [39]. Employing serum creatinine elevation as a diagnostic criterion for acute kidney failure, considering that basal creatinine levels in the patients included in our study were near 4 mg/dL, would not be adequate, as serum creatinine in these stages of kidney disease is more a marker of nutritional status than of kidney function. However, we decided to use two cut-off points for serum creatinine levels to assess renal function impairment. The first, an elevation greater than 0.3 mg/dl, a cut-off point with high sensitivity, to detect differences in creatinine elevation between both groups, as was the case in the study. The second, a double elevation of baseline creatinine levels, a cut-off point with high specificity that could be more related to a clinically significant elevation, which did not occur in any of the groups.

One of the main adverse effects reported in the literature associated with thiazide use is hydroelectrolytic imbalance, hyperuricemia or an increased diabetes risk [40–42]. Our results did not show any significant differences in uric acid risk (there was no gout attack in any group), blood glucose, potassium or sodium levels at the end of follow-up. There was one case of acute myocardial infarction in the intervention group, which required endoprosthesis insertion via catheterization. Cardiovascular function improved significantly following intervention; however, kidney function remained unchanged, with final GFR readings similar to baseline.

This study has some others limitations. First, the follow-up in our study was short; therefore, further prospective trials are needed to assess the long-term safety and efficacy of this regimen. Second, there was patient noncompliance with dietary recommendations, even though all patients were evaluated and counseled in terms of renal nutrition, with an emphasis on sodium dietary restriction, as it is known that sodium excretion is directly proportional to kidney function and sodium dietary intake [43]. Third, this trial was done at a single center which could limit generalizability. Fourth, this study does not include hard clinical outcomes, which may be interesting to investigate in future studies. Finally, most of our patients did not have a basal echocardiogram at the time of enrollment. Having an estimate of ventricular function beforehand may have allowed us to identify the presence of cardiac failure or changes in left ventricle ejection fraction or telediastolic volume, which may have explained the observed BNP behavior.

In conclusion, our study demonstrated the efficacy of chlorthalidone and bumetanide on volume overload and blood pressure reduction in advanced chronic kidney disease patients. Our results suggest that at the one-month follow-up, bumetanide plus chlorthalidone therapy is effective in patients with advanced chronic kidney disease. Volume overload reduction was adequately tolerated in this population; however, further studies with larger samples and longer follow-up periods are warranted. Based on this trial and those conducted previously on thiazide use for advanced chronic kidney disease, we propose that current guidelines should be reviewed to perhaps change the notion that thiazides should not be used in advanced CKD patients, as it has been demonstrated that this class of diuretics can be helpful in the treatment of this population.

## Data Availability

The datasets used and analyzed during this study are available from the corresponding author on reasonable request.
